# Some Past Incidents in the Life of the Dental Register

**Published:** 1900-03-15

**Authors:** 


### SOME PAST INCIDENTS IN THE LIFE OF THE DENTAL REGISTER.

BY THE EDITOR.

(*Continued from January No., page* 38.)

Number 1 of Volume X. began with an issue published in October, 1856. Dr. J. Taft and Dr. George Watt were the editors. This number consisted of 152 pages and ten pages of advertising. In an editorial the editors promise to enlarge the REGISTER as soon as the subscription list will justify. They also state that as soon as arrangements can be made for it, that it will be issued a month earlier to avoid being issued at the same date as other journals, and thus furnish the profession journal reading in installments. The next number was issued in December instead of January.

The first number published under Taft and Watt's management contains several original articles, and the proceedings of the second annual meeting of the American Dental Convention, held in New York City, August 6, 7 and 8, 1856. The same number contains the proceedings of the sixteenth annual meeting of the "American Society of Dental Surgeons," August 5, 1856, at New York City, which failed after two unsuccessful efforts to obtain a quorum. The secretary managed to secure a sufficient attendance to have his minutes approved and to hear the following report of a committee appointed at the previous session. This committee reported that it had given the question of dissolving the organization known as the "American Society of Dental Surgeons," the consideration which its importance demanded, and would report this resolution: *Resolved*, That we deem it expedient to dissolve this Association, and that it be and is hereby adjourned *sine die*. Signed, Elisha Townsend, E. J. Dunning, John S. Clark and R. Arthur. The resolution was adopted and the funds ordered distributed in accordance with the Constitution.

More than four-fifths of the space in the first number is given up to reports of dental society meetings, and they are very good reports, too, more readable than some more modern productions. The report of the second annual meeting of the "Western Dental Society," at Chicago, July 30, 1856, is very interesting. Dr. Kennicott at this meeting suggested the use of a hickory plug for extracting the pulp of a tooth.

According to the report of the second annual meeting of the American Dental Convention, held in New York City, there were present nearly two hundred delegates, including two from Cuba. One of the first acts of the session was to vote that the two sections of the Constitution conflicting with a resolution introduced to elect every one present a member who was willing to sign the Constitution be repealed. By this action several persons who were not practitioners of dentistry and who never had been were made members. Drs. Neal, McQuillen and Dwinell, who championed the action, stated that they wished to make the organization the simplest possible that could keep a body of men together. We have since learned wisdom.

They had a curious method of transacting business in that convention. A subject for discussion was announced and volunteers were called for to open the discussion.

After every one had said all he cared to, the essayist read a carefully prepared paper on the subject, and then this paper was discussed. We suppose the idea was to lead up to the subject gradually. One distinguished gentleman claimed to have seen, through the microscope, the nerves of the tooth extending from the pulp through the dentin, and in some cases half way through the enamel. Another gentleman asserted that the dentin contained tubuli, but that he was satisfied from his microscopical researches that they were empty. Another gentleman stated that he always found sensitive dentin associated with disorder of the digestive tract and he put such patients on a cathartic treatment, lasting from three days to three months.

At this meeting two plates made of vulcanized rubber were shown, but it was stated that the process could not be used because of patents existing on the process of hardening the rubber.

It seems from an editorial in the next issue of the REGISTER that there was considerable dissatisfaction with this report of the convention. It was made by a special reporter employed by the convention and his report was edited by a committee and then put in type and stereotyped and the plates sent to each of the dental journals. The editorial comment, after explaining how it happened, says: "Now, gentlemen, if you are misrepresented, you have the consolation to know that you have misrepresented yourselves, and while it misrepresents some of you by making your speeches shorter, and worse, it does equal or greater injustice to others, by making theirs lengthier and better than they were. The former can, at the next meeting, make an advance on the reputation acquired by the published reports of the meeting; while the latter must, by gigantic efforts, sustain their acquired reputations, or be regarded as all birds are which fly high and light low. We do not intend by these remarks to reflect on the reporter.

The probability is that some of the speeches improved more than others during the period of incubation in which the committee sat on them. It is perfectly natural that *some* of them should. They all had time to grow; and it is not unnatural that those indigenous to the soil should make the most rapid growth."

This intimation of unfaithfulness or unreliability detracts much from the full enjoyment which comes from reading these reports of the early efforts made to carry on dental societies, but at the same time it more indelibly stamps on the historic page the spirit of the time, and which true and noble men had to combat and overcome to give us the privileges we to-day enjoy in the truly benevolent sentiment which everywhere possesses professional men and makes it possible for us to call ours a liberal and benevolent profession, able to support many institutions and associations, all striving to do their utmost to enlarge our sphere of usefulness. The dental journal of to-day is not the least important of these institutions.

The following interesting resolution is taken from the proceedings of the Western Dental Society at a meeting held in St. Louis, May 20, 1857: "*Resolved*, That to Dr. Arthur, and he alone, the dental profession is under obligation for his liberality in laying before the profession the principle of using and welding together annealed gold by the use of serrated pointed instruments, and this Society desires to express its thanks to him for this real improvement in the mode of operating. Signed, H. J. McKellops, J. Taft and W. W. Allport."

One of the editors attended this meeting of the Western Dental Society, and in an editorial comment makes the following interesting statement: "Quite a number of new members were added to the society, and from the length of time occupied by the examining committee with the candidates, and from their appearance after they were through, we rather surmise that they thought the way into this society was a hard road to travel. Unless you are about right in that which constitutes the man—integrity and good intentions, also professional attainments—you need not knock at the door of the Western Dental Society for admission." This is quite in contrast with the ideas of the *American Dental Convention*.

The following extracts from an editorial in the last number of the volume, gives a summary of the experience of the new editors and indicates that possibly the editorial life in those days was not one of unalloyed pleasure, such as it now is to some of us fortunate modern caterers:

"A year has elapsed, and this number closes a volume. A short experience, says one. But, hold on—we wish to do all the talking in this meeting, and, after it is adjourned, you can, if you like, hold one of your own, and will not interrupt you. We did not intimate that we had had a long experience; but, sometimes, we consider it a *strong* one."

"Well, we set out full of hope and high aspirations. We promised to enlarge the REGISTER when you had given us the necessary aid in extending its circulation, and we really thought you would do that right off, and we (that is, I, for our comrade' don't get excited), lay awake nights thinking of the DENTAL REGISTER, Vol. XI., a quarterly journal of 160 pages—but there is no use in telling these symptoms now, for we have got entirely over that complaint!"

"You know—you ought to—that our terms are 'in advance.' With the second number we sent bills to such as had not remitted, with the result that within the month about *one subscriber in thirty* remitted. With a subscription list that would make us feel rich if each would send the $3 in advance, we have so far received about half the cost of publishing. Well, do you think we are discouraged?

Nothing of the kind. Our hopes are not so buoyant as a year ago, but better balanced. We know the REGISTER has some *friends;* and when we see people enjoying a very comfortable sleep, we expect great energy when they awake. And we hope soon to receive not only last year's subscription, but $6 in payment for Volume XI. We know, too, that the REGISTER is a fixed fact, and will live, even should we die at our posts."

"We find that the more we labor for the more attached we become to our profession, and no labor is to us more pleasant than that bestowed on the REGISTER. We trust that, through your co-operation and counsel, this labor may be properly directed."

This editorial seems to have made some impression, for in the next number is published a list of nearly one hundred who sent the $3 for Volume X. Eight paid for Volume XI. and one paid for Volume XII.

(*To be Continued.*)

